# Medication histories documentation at the community pharmacy setting: A study from Jordan

**DOI:** 10.1371/journal.pone.0224124

**Published:** 2019-10-22

**Authors:** Rana Abu Farha, Khawla Abu Hammour, Tareq Mukattash, Raja Alqudah, Rand Aljanabi

**Affiliations:** 1 Department of Clinical Pharmacy and Therapeutics, Faculty of Pharmacy, Applied Science Private University, Amman, Jordan; 2 Department Biopharmaceutics and Clinical Pharmacy, Faculty of Pharmacy, The University of Jordan, Amman, Jordan; 3 Department Clinical Pharmacy, Faculty of Pharmacy, Jordan University of Science and Technology, Irbid, Jordan; Newcastle University, UNITED KINGDOM

## Abstract

**Objectives:**

The main objective of this study was to evaluate community pharmacists’ awareness and perception about medication reconciliation service and to assess the completeness of collecting patients’ medication histories in the community pharmacy setting.

**Methods:**

A cross-sectional study was conducted between February to March 2018 in Amman-Jordan. During the study period, 150 community pharmacists were invited to participate in the study. Each pharmacist completed a validated structured questionnaire evaluating their awareness, current practice, perceived attitude and perceived barriers towards the implementation of medication reconciliation and the collection of medication histories at the community pharmacy setting.

**Results:**

A total of 121 pharmacists agreed to participate and filled the questionnaire. Our results showed that only 13.2% of the pharmacists were able to define “medication reconciliation” correctly, and around 31% have a misconception that the medication reconciliation process should be performed only at the inpatient setting.

Only 19.8% (n = 24) of the participating pharmacists stated that they ask all patients for a complete current medication list of medications when they arrive at the pharmacy site. Medication histories for most patients were lacking information about the dosage, route, frequency, and time of the last refill for each medication listed. “Patients lack of awareness about all the medications they are receiving” was the main barrier discouraging community pharmacists from collecting medication histories and participating in reconciliation service.

**Conclusion:**

Community pharmacists in Jordan showed a low awareness about the medication reconciliation concept and demonstrated a modest role in obtaining medication histories in community pharmacies. But still, they showed a positive attitude towards their role in implementing the different steps of medication reconciliation. This suggests that educational workshops to increase pharmacists’ awareness about their role and responsibilities in collecting a complete and accurate medication history are warrented.

## Introduction

Medication errors represent one of the most common causes of morbidity and mortality in the hospital settings [1, 2]. They can occur from the first step of visiting a physician clinic until the last one of taking medicines; they include incorrect drug administration, prescribing errors, dose miscalculations, failed communication and lack of patient education [3–5].

Among the most commonly encountered medication errors are discrepancies occurring upon transferring of patient between different sites of care [2, 6, 7]. Poor communication of patients’ medication histories between community practitioners and hospital practitioners may contribute to such discrepancies [8]. It was found that more than 67% of patients have at least one discrepancy in their medication histories at the time of their hospital admission [8].

One of the most important methods, which have been proved for reducing medication errors, is medication reconciliation [8–10]. Medication reconciliation is defined as “the process of optimizing the list of all medicines administered by the patient, including the name, dosage system, frequency and the route of each medication, and using this accurate prescription to provide the correct treatment for the patient” [11, 12]. Joint Commission in 2006 projected medication reconciliation as an effective process to prevent those medication errors [13]. It ensures that healthcare providers will have an updated list of patients’ medications, thus, it has the potential to improve patients’ safety and ensure optimal care for them.

Many published studies made clear evidence on the beneficial value of medication reconciliation in reducing medication errors [6, 14–16]. It has been estimated that 50–90% of medication errors were reduced in United States hospitals due to medication reconciliation [7, 17, 18]. This was achieved through engaging healthcare providers into workshops and seminars on how to reduce medication errors and adverse drug reactions and how to improve medication safety [7]. Also in 2014, the World Health Organization (WHO) the Standard Operating Protocol (SOP) to assure medication accuracy at transitions in care and it encouraged all its member countries to promote the implementation of the medication reconciliation SOP [19]. Despite the clear role of medication reconciliation in decreasing medication errors [20], the success of this process depends on the ability of hospital personnel to implement the first step in medication reconciliation which involve the collection and access of the most updated medication histories (i.e the Best Possible Medication History (BPMH)). The BPMH should be created and collected within 24 hours of hospital admission [21]. The BPMH reflects an accurate and complete list of all medications taken by the patient before admission, and this list should be confirmed by multiple sources, among them is the community pharmacy records. Obtaining medication history is considered the first step in assessing discrepancies and it is a crucial step in medication reconciliation [22].

Several studies have shown that obtaining the BPMH is important and can be done by pharmacists effectively [23–26]. Besides, pharmacists are suited to conduct medication reconciliation and to obtain the BPMH as they are more familiar with drug names, frequency, and dosage forms [26]. Also, any inconsistencies and mistakes in patients’ self-reported medication histories can be identified by pharmacists regardless of the setting. This was clear from a study that compared the process of obtaining patient BPMH by a pharmacist versus physician, where researchers found a significantly higher number of medications identified by pharmacists compared by those identified by physicians [26]. Also, physicians documented less medication doses and dosage schedules than did pharmacists [26]. Also, several studies have confirmed that when pharmacists obtained patients’ medication histories, more medication discrepancies were detected [23–25].

Thus, by highlighting the importance of collecting the BPMH in providing medication reconciliation, pharmacists working in community pharmacies must be able to develop a process to obtain a complete and correct patient’s medication history at the community setting, thus supporting the available sources that can be used in collecting the BPMH at the hospital setting. Thus, the main objective of this study was to evaluate community pharmacists’ perceptions of medication reconciliation and their role in collecting medication histories as an essential source to collect patients’ BPMH. Also, we aimed to assess the completeness of collecting patients’ medication histories and to identify barriers that prevent them from obtaining appropriate medication information.

## Material and methods

### Study design, settings and study subjects

Between 16^th^ February- 14^th^ March 2018, a cross-sectional survey-based study was conducted on pharmacists working at community pharmacies in Amman-Jordan. During the study period, 150 community pharmacists were invited to participate in the study using a self-administered survey. Before distributing the surveys, an informed verbal consent was obtained from pharmacist after being informed about the purpose and instruction of the study. The purpose and instruction of the study were presented on the front page of the questionnaire to ensure that the same information was delivered to all participants. Once participants agreed verbally to participate, the questionnaires were distributed to them to be filled out, and they were informed that their participation was voluntary and their responses would be kept anonymous.

### Sample size calculation

Based on a study conducted in the United States to evaluate the awareness of medication reconciliation in the community pharmacy setting, with 65% of pharmacists were aware about the service [27], and using a desired precision of 10%, and 95% level of confidence, a minimal sample size of 88 pharmacists was considered to be representative for this study.

### Ethical consideration

The study was performed following the ethical protocols outlined in the World Medical Association Declaration of Helsinki guideline [28]. Ethical approval was obtained before conducting the study from the Institutional Review Board (IRB) at the Jordan University Hospital (Reference number: 65/2017).

### Questionnaire development

Questions involved in the study questionnaire were drawn from a previous study that has evaluated pharmacists’ awareness about medication reconciliation in the community pharmacy setting [27]. The final draft of the questionnaire was pilot tested by a target of five pharmacists. Based on their feedback, some amendments were performed to enhance the clarity of the questions and to ensure the comprehension of the tool. The final version of the questionnaire is divided into five domains of interest including:

1. pharmacists’ demographic characteristics (9 questions) including pharmacists’ gender, age, country of graduation, year of graduation, their experience as a pharmacist, educational level, site of work, current job responsibilities and the number of patients served per day during their working hours.
2. Pharmacists’ knowledge about medication reconciliation service (2 questions). In this domain, the first question was an open-ended question where pharmacists were asked to define medication reconciliation, and the other question was a close-ended.
3. Assessment of medication histories collected at the community pharmacy site (7 questions). All questions in this domain were close-ended questions.
4. Pharmacists’ perceived attitude towards medication reconciliation (8 statements). In this domain, a Likert scale of 5 (strongly agree (5), agree (4), neural (3), disagree (2), and strongly disagree (1)) was used to evaluate the level of agreement for each statement.
5. Pharmacists’ perceived barriers towards the implementation of medication reconciliation (5 statements). Also here, a Likert scale of 5 (strongly agree (5), agree (4), neural (3), disagree (2), and strongly disagree (1)) was used to evaluate the level of agreement for each statement.

### Statistical analysis

Data were analyzed using statistical package for social science (SPSS) version 22 (SPSS Inc., Chicago, IL, USA). The descriptive analysis was done using mean/standard deviation (SD) for quantitative variables and frequency/percentage for categorical variables.

## Results

### Socio-demographic characteristics of pharmacists

Among the 150 pharmacists invited to participate in this study, a total of 121 agreed to participate and filled the questionnaire (response rate = 80.7%). The majority of pharmacists were female (n = 94, 77.7%) and had a Bachelor degree in pharmacy (BPharm/PharmD) (n = 104, 86.0%). Summary of demographic characteristics of participating pharmacists is summarized in Table 1.

**Table 1:** Demographic characteristics of the study sample (n = 121).

Parameters	Mean (SD)	n	%
***Age (years)***	28.8 (7.4)		
***Gender***			
• Male		27	22.3
• Female		94	77.7
***Experience as a pharmacist***	5.3 (6.3)		
***Educational level***			
• BSc (BPharm/PharmD)#		104	86.0
• Graduate studies (MSc/PhD)		17	14.0
***Country of graduation***			
• Jordan		118	97.5
• Others		3	2.5
***Site of work***			
• Independent community pharmacy		58	47.9
• Chain community pharmacy		63	52.1
***Current job responsibilities***			
• Managerial		23	19.0
• Counselling & dispensing		83	68.6
• Clinical Pharmacy		8	6.6
• Drug Information		7	5.8
***Number of patients visited pharmacy/day***			
• Under 50		49	40.5
• 50–99		37	30.6
• 100–149		21	17.4
• 150–199		9	7.4
• 200 or above		5	4.1

^#^In Jordan both BPharm (the Bachelor in pharmacy) and Pharm D (Doctor of pharmacy) are classified as Bachelor degree by the ministry of higher education

### Pharmacists’ knowledge about medication reconciliation service

Fifty-eight percent of pharmacists (n = 71) revealed that they were familiar with the term “medication reconciliation”, yet only 13.2% (n = 16) of them were able to define it correctly.

### Medication history collection at community pharmacy site

Table 2 lists pharmacists’ responses to questions about medication histories collection at community pharmacy sites. From the table, it is evident that only 19.8% (n = 24) of the participating pharmacists stated that they ask all patients for a complete current medication list when they arrive at the pharmacy site. Also, only 13.2% (n = 16) of the pharmacists said that more than 50% of patients have a complete current medications list at their pharmacy site.

**Table 2:** Pharmacists BPMH collection at community pharmacy site (n = 121).

Question	Pharmacists response	
	n	%
***Do you routinely ask patients for a complete CURRENT list of medications when they arrive in your pharmacy*?**		
• Yes, for all patients	24	19.8
• Yes, but only for selected patients	88	72.7
• Rarely	7	5.8
• Never	2	1.7
***What percentage of patients has a CURRENT and COMPLETE list of their medications when they arrive in your pharmacy*?**		
• Less than 10%	37	30.6
• 11% to 20%	21	17.3
• 21% to 30%	29	24.0
• 31% to 50%	18	19.4
• Greater than 50%	16	13.2
***Is your pharmacy computer system capable of printing a list of CURRENT prescription medications for patients on demand*?**		
• Yes	74	61.2
• No	47	38.8
***Does the computer-generated medication list provided to patients include the dosage*, *route*, *frequency*, *and time of last refill for each medication listed*?**		
• Yes	37	30.6
• No	52	34.0
• Not applicable	32	24.6
***Does your pharmacy computer system have the ability to document*, *as part of the patient’s CURRENT medication profile/list*, *OTC drugs*, *and herbal products*?**		
• Yes	38	31.4
• No	55	45.5
• Not applicable	28	23.1
***Does your pharmacy routinely provide a list of CURRENT medications to patients*?**		
• Yes	38	68.6
• No	83	31.4
***Did your pharmacy have a policy to provide hospitals with CURRENT list of medications for each patient that enters the Emergency Department or is admitted*?**		
• Yes	21	17.4
• No	100	82.6

When pharmacists asked about their pharmacy computer system, 61.2% (n = 74) said that their system is capable of printing a list of patients’ current prescription medications on demand. Among those computer systems, only 30.6% (n = 37) of them include information about the dosage, route, frequency, and time of the last refill for each medication listed, and 31.4% (n = 38) can document OTC and herbal products in addition to prescription medications. Despite the availability of the current medication list on the pharmacy computer system in 61.2% of involved pharmacies, only 31.4% (n = 38) of respondents said they routinely provide a list of medications to patients.

Regarding sharing of medication information, only 17.4% (n = 21) of respondent confirmed the availability of policy within their pharmacies to provide hospitals with medications list for each patient enters the emergency department or upon hospital admission.

### Pharmacists perceived attitude towards medication reconciliation service

Statements to assess pharmacists’ perception of medication reconciliation divided into six positive statements and two negative statements (Table 3). Around one-third of the pharmacists (n = 38, 31.4%) have a misconception that the medication reconciliation process should be performed only at the inpatient settings and it does not apply to the community settings and that only prescription medication list should be evaluated during the reconciliation process.

**Table 3:** Pharmacists perceived attitude towards medication reconciliation (n = 121).

Statement	Pharmacists’ responses n (%)				
	Strongly agree	Agree	Neutral	Disagree	Strongly disagree
The medication reconciliation process should be performed only at the inpatient settings and it is not applicable at the outpatient settings.	10 (8.3)	28 (23.1)	24 (19.8)	39 (32.2)	20 (16.5)
The medication list that should be evaluated during reconciliation process is limited to prescription drugs only.	9 (7.4)	29 (24.0)	30 (24.8)	29 (28.1)	9 (15.7)
Pharmacists can help in assessing the appropriateness of medications for patients	62 (51.2)	54 (44.6)	2 (1.7)	3 (2.5)	0 (0.0)
I am confident that I can provide advice for healthcare providers about the medications that are prescribed for patients	48 (39.7)	64 (52.9)	8 (6.6)	1 (0.8)	0 (0.0)
Pharmacists should be consulted for advice when medical staff are considering stopping or prescribing medications	59 (48.8)	55 (45.5)	3 (2.5)	2 (1.7)	2 (1.7)
Pharmacists can counsel and educate patients, families and nursing staff about medications prescribed	72 (59.5)	44 (36.4)	4 (3.3)	1 (0.8)	0 (0.0)
Pharmacists can help the doctor in identifying suitable formulations of medications	64 (52.9)	52 (43.0)	3 (2.5)	2 (1.7)	0 (0.0)
Pharmacists must be integral members of the care team to provide services for patients	63 (52.1)	51 (42.1)	5 (4.1)	1 (0.8)	1 (0.8)

Pharmacists felt positively towards their role in providing medication reconciliation and counseling services to patients. More than 90% of respondents believed that they can help in assessing the appropriateness of medications for patients, that they can provide advice for healthcare providers about the medications prescribed for patients, can offer advice when medical staff are considering stopping or prescribing medications, can counsel and educate patients, families and nursing staff about medications prescribed, and they can help the doctor in identifying suitable formulations of medications. Also, pharmacists felt that they must be integral members of the care team to provide services for patients (n = 114, 94.2%).

### Pharmacists perceived barriers against collection of medication histories at community pharmacy practice sites

Barriers that may discourage community pharmacists from collecting medication histories are presented in Table 4. The main barrier was ‘‘Patients lack awareness about all the medications they are receiving”, where 80.1% (n = 97) agreed/strongly agreed. Lack of communication between pharmacists and healthcare providers was the second most important barrier (n = 96, 79.3%).

**Table 4:** Pharmacists perceived barriers against the collection of BPMH at community pharmacy practice site (n = 121).

Statement	Pharmacists’ responses n (%)				
	Strongly agree	Agree	Neutral	Disagree	Strongly disagree
Lack of knowledge in medication reconciliation among pharmacists	27 (22.3)	67 (55.4)	19 (15.7)	8 (6.6)	0 (0.0)
Patients not aware of all the medications they are receiving.	32 (26.4)	65 (53.7)	17 (14.0)	7 (5.8)	0 (0.0)
Handwritten or printed medication lists that patients or their caregivers supply are incomplete.	28 (23.1)	64 (52.9)	24 (19.8)	5 (4.1)	0 (0.0)
Lack of communication between pharmacists and healthcare providers	35 (28.9)	61 (50.4)	16 (13.2)	7 (5.8)	2 (1.7)
Lack of time to perform a complete review of the patient’s medications.	41 (33.9)	47 (38.8)	21 (17.4)	8 (6.6)	4 (3.3)

## Discussion

Obtaining an accurate and complete medication history (the BPMH) is an essential first step of the medication reconciliation process, to ensure patient safety and an effective medical practice [29]. A variety of sources can be used to obtain the BPMH and accommodate the medication reconciliation process. Direct interviews with patients or their caregivers, healthcare providers interview, community pharmacy records review, previous patient’s health records, patient medication vials all of this can be used as medication histories sources to support the BPMH [19]. Usually, more than two resources should be used [30], since each source has its limitations, and none can stand alone as a complete and accurate source [19].

Community pharmacy records can be a useful source for obtaining medication histories, where 80% of patients usually use the same pharmacy to refill their regular medications [31]. Also, community pharmacists are an ideal source for obtaining medication histories and assisting the reconciliation process [32]. They are capable of obtaining medication histories characterized by high accuracy, thus produce fewer medication errors [32]. The completeness and accuracy of the different medication histories sources were evaluated in a previous study, which showed that community pharmacy records were among the least complete sources for medication histories [32].

Our results showed that only 58% of the community pharmacists were familiar with the term medication reconciliation, but only 13.2% of them defined the term correctly. The image on the current practice of medication reconciliation is not clear in Jordan; that is why many healthcare providers were not aware of the medication reconciliation concept. This was evident in a previous study by Abu Hammour et al, which showed a low level of awareness and understanding about the medication reconciliation process among Jordanian hospital pharmacists [33]. Low awareness about medication reconciliation in Jordan may be due to the lack of training workshops, scientific meetings or conferences, the lack of university curricula focusing on the extra roles that can be played by the pharmacists. In assessing the practice of obtaining medication histories at the community pharmacy setting, only 19.8% of the participating pharmacists stated that they ask all patients for a complete current medication list when they arrive at the pharmacy site. Also, only 13.2% of the community pharmacists said that more than 50% of patients have a complete current medications list at their pharmacy site. A previous study reported a similar findings, where study researchers have evaluated the completeness of different information resources used to obtain BPMH at the emergency department for pediatric patients, and they found that information from community pharmacies was available for only 24% of the admitting patients [30]. Also, in a study conducted in the United States, 46% of the pharmacists stated that they rarely ask patients for a current list of medications when they arrive in the pharmacy [27]. This is not surprising and expected since the concept of medication reconciliation is a new concept introduced a few years ago within the pharmacy profession [26].

The study has also shown that despite the availability of the current medication lists at the pharmacy computer system in 61.2% of the involved pharmacies, only 31.4% of the respondents said that they routinely provide a list of medications to patients. In comparison with Horn and Gaunt’s study, less than 50% of respondents stated that they routinely provide a medication list to patients when they visit the pharmacy [27]. The result of this study support the idea that community pharmacists don’t frankly understand their roles and responsibilities at the different steps of medication reconciliation. Thus, we need to enhance community pharmacists awareness about their roles and responsibilities at each step of medication reconciliation to optimize the implementation of the process [34].

Among the pharmacy computer systems, only 30.6% include information about the dosage, route, frequency, and time of the last refill for each medication listed. A higher percentages were reported in two previous studies where Horn and Gaunt found that 59% of computer systems contain information regarding dose and frequency [27], while Dersch-Mills et al found that the median completeness score of community pharmacy records regarding the dose and frequency was around 33% (IQR 4% to 56%) [30].

It was found that around one-third of the community pharmacists (n = 38, 31.4%) have a misconception that the medication reconciliation process should be performed only at the inpatient settings and does not apply to the community settings and that only prescription medication list should be evaluated during the reconciliation process. Although the number of community pharmacists who believed that medication reconciliation could be applied in the community setting considerably is low; they showed a positive attitude towards their role in providing medication reconciliation and counseling services to their patients. Adding to that, more than 90% of respondents believed that they could help in assessing the appropriateness of medications for patients and applying the process of reconciliation.

It worth saying that community pharmacists have an essential role in providing medication reconciliation at two transition point, firstly at hospital admission by providing a reliable source of medication histories to the hospital personnel, secondly at hospital discharge through identifying medication discrepancies between the hospital medication order and the discharge medication dispensed at the community pharmacies [23, 26, 35, 36].

In our study, the main barrier that discouraged the community pharmacists from collecting medication histories was patients’ lack of awareness about all the medications they were receiving. Lack of communication between community pharmacists and healthcare providers is the second most important barrier. In Horn and Gaunt’s study, the main barrier against practicing medication reconciliation was the low frequency of communication between healthcare providers and community pharmacists [27]. Other barriers that may affect the completeness and accurateness of medication histories were time constraints, the lack of patient’s familiarity with his or her medications, and the severity of the patient’s illness [22]. It is important to clarify that despite the presence of such barriers against the implementation of medication reconciliation and the collection of medication histories, but with practice and improving skills, the process may become easier [7].

Finally, we are aware of a methodological limitation of this study; as the study relied on the self-administered questionnaire, which may overestimate community pharmacists’ awareness and practice regarding the medication histories collection and the reconciliation process.

## Conclusion

In conclusion, this study demonstrated the low awareness about the medication reconciliation concept among community pharmacists and revealed the modest role played by them in obtaining medication histories at the community pharmacy setting. But still, community pharmacists showed a positive attitude towards their role in implementing the different steps of medication reconciliation which could help in assessing the appropriateness of medications for patients.

Such findings suggest the need to increase community pharmacists’ awareness about their role and responsibilities in collecting complete and accurate medication histories.
